# 2318. A Composite Peptide Vaccine Comprised of Conserved SARS-CoV-2 and Influenza Epitopes Generated Antisera Reponses to Both Coronavirus and Influenza

**DOI:** 10.1093/ofid/ofac492.149

**Published:** 2022-12-15

**Authors:** Nimisha Rikhi, Kevin Muema, Kellie Kroscher, Richard F Schuman, Aba Assiaw-Dufu, Clara J Sei, Gerald W Fischer

**Affiliations:** Longhorn Vaccines & Diagnostics, LLC, Gaithersburg, Maryland; Longhorn Vaccines & Diagnostics, LLC, Gaithersburg, Maryland; Longhorn Vaccines & Diagnostics, LLC, Gaithersburg, Maryland; Antibody and Immunoassay Consultants, Rockville, Maryland; Longhorn Vaccines & Diagnostics, LLC, Gaithersburg, Maryland; Longhorn Vaccines & Diagnostics, LLC, Gaithersburg, Maryland; Longhorn Vaccines & Diagnostics, LLC, Gaithersburg, Maryland

## Abstract

**Background:**

While influenza and SARS-CoV-2 virus can be severe, co-infection with these viruses has the potential for worsening the clinical outcome. The combined infection of influenza A virus (IAV) and SARS-CoV-2 in animal models has also shown elevated SARS-CoV-2 viral load and pneumonia. Co-vaccination may provide a useful option in prevention of combined infection. In this study, a composite peptide vaccine comprised of highly conserved influenza neuraminidase (NA) and matrix protein (M2e) epitopes, in combination with either conserved spike protein (SP) or RNA polymerase (POL) epitopes of SARS-CoV-2 generated robust immune responses to both coronavirus and influenza virus.

**Methods:**

Mice were immunized with 20µg of Coronavirus Pep02 (CorPep02, comprised of POL) or Coronavirus Pep05 (CorPep05, comprised of POL and NA+M2e) or Coronavirus Pep11 (CorPep11, comprised of SP and NA+M2e), formulated with AddaVax™. Serum antibody titers to both coronavirus and influenza peptides, and whole viruses were analyzed using ELISA. Antisera neutralizing activity against IAV was determined using microneutralization assay (MNA).

**Results:**

CorPep05 immunization generated enhanced IgG1 and IgG2b antisera responses to the immunogens compared to CorPep02, starting at Day-21. Immunization with CorPep05 or CorPep11 induced strong IgG1 and IgG2b immune responses to the composite coronavirus peptides, influenza NA and M2e peptides, coronavirus POL and SP peptides. Antisera from CorPep05 and CorPep11 groups bound to both viruses (IAV and human coronavirus NL-63 (HCoV NL-63)) and demonstrated neutralizing activity against IAV, with titers >5000.

**Conclusion:**

A composite peptide vaccine comprised of both influenza (NA+M2e) and coronavirus (POL) epitopes generated enhanced immune responses in comparison to peptide containing only POL. Th1 and Th2 immune responses to coronavirus POL and SP were observed. Serum neutralizing activity was demonstrated against IAV. Studies are underway to examine neutralizing antibodies against HCoV NL-63 and multiple variants of SARS-CoV-2. Highly conserved epitopes in these composite peptide vaccines may provide an important strategy to prevent infections with IAV or SARS-CoV-2 and mitigate the threat of co-infection.

**Disclosures:**

**All Authors**: No reported disclosures.

